# Episomal reprogramming of Duchenne muscular dystrophy patients derived CD3^+^ T cells towards induced pluripotent stem cells

**DOI:** 10.12669/pjms.37.2.3388

**Published:** 2021

**Authors:** Mehwish Zehravi, Mohsin Wahid, Junaid Ashraf

**Affiliations:** 1 Mehwish Zehravi, Dow Research Institute of Biotechnology and Biomedical Sciences, Dow University of Health Sciences, Karachi, Pakistan; 2 Mohsin Wahid, Dow Research Institute of Biotechnology & Biomedical Sciences and Department of Pathology, Dow International Medical College, Dow University of Health Sciences, Karachi, Pakistan; 3 Junaid Ashraf (Rtd) Department of Neurosurgery, Dow University of Health Sciences, Karachi, Pakistan

**Keywords:** Induced pluripotent stem cells, Episomal reprogramming, Duchenne muscular dystrophy

## Abstract

**Objective:**

To derive Duchenne muscular dystrophy patient specific induced pluripotent stem cells (iPSCs) from CD3^+^T cells of DMD patients using episomal reprogramming and characterization of these DMD-iPSCs using immunofluorescence to confirm their pluripotent state.

**Methods:**

DMD patients were selected based upon their clinical history and examination. Peripheral blood mononuclear cells were isolated from peripheral blood of DMD patients (n=3) by density gradient centrifugation and were used to generate DMD patient specific T cells (DMD-T cells) using rhIL-2, plate bound anti CD3 antibody and T cell specific media along with specific culture conditions that promote T cell expansion. CD3^+^ T cells were characterized by flow cytometry and reprogrammed using episomal plasmid to generate DMD-iPSCs. These DMD-iPSCs were characterized using immunofluorescence. The study was carried out at Dow Research Institute of Biotechnology and Biomedical Sciences during August 2017- July 2018 for a period of approximately 12 months.

**Results:**

The peripheral blood mononuclear cells (PBMNC) derived T cells appeared as suspended cell clumps morphologically were positive for the expression of CD3 and negative for CD31. The DMD patient specific iPSCs appeared as round, compact and flat colonies with well-defined edges morphologically and were positive for the expression of pluripotency markers OCT4, SSEA-4 and TRA-1-81 on immunofluorescence.

**Conclusion:**

CD3^+^ T cell derived DMD-iPSCs were obtained under feeder free and xeno-free culture conditions using episomal reprogramming.

## INTRODUCTION

Duchenne muscular dystrophy (DMD) is a lethal disorder with out of frame mutations in dystrophin gene which impairs the production of dystrophin protein responsible for muscle strength and contractility. It is inherited in an X linked recessive manner and particularly affects boys. Deficiency of dystrophin protein results in inefficient linkage between intracellular actin and extracellular laminin resulting in muscle fragility, inefficient contractile ability, contraction induced damage, necrosis and inflammation at the site of damage. DMD is fatal amongst all muscular dystrophies. The muscle weakness begins early between two to five years of age and the child becomes wheel chair bound by 10 - 14 years of age. These children suffer from respiratory and cardiac complications during the second decade of life. Death occurs around second or third decade due to left ventricular dysfunction and respiratory failure.^1^

The development of stem cell research has been a major breakthrough in the field of biomedical research. Induced pluripotent stem cells (iPSCs) are obtained by genetic reprogramming of adult cells and tissues to an embryonic stem cell like state by forced expression of several genes and transcription factors. Yamanaka proposed the concept of induced pluripotency to generate stem cells similar to embryonic stem cells without utilizing egg or embryo thus avoiding ethical concerns. This revolutionary experiment was first performed in mice in 2006 and later was replicated in humans using fibroblast as cell source.^2^,^3^ The major shortcoming of both these experiments was the requirement of surgical intervention for isolating human fibroblast which makes it impractical source for reprogramming. In contrast to it, T cell obtained by peripheral blood is a most suitable approach to derive induced pluripotent stem cells as venipuncture is a non-invasive technique and is routinely carried out as a blood sampling procedure. In our study we have used T cells as a cell source for generating iPSCs. T cells are comparatively abundant in peripheral blood (6.5 x 10^5^ - 3.1 x 10^6^/ml).^4^ Using the T cells as a cell source for generation of induced pluripotent stem cells would circumvent the issue of obtaining punch biopsies from patients.

Integration free iPSCs derived by episomal reprogramming are comparatively safer alternative and can serve as disease models and as clinical grade cells for cell therapies in future. Integration free reprogramming is also referred to as foot print free reprogramming. Since it is devoid of genomic integration there is a lower incidence of genomic aberrations and the safety concerns with the use of viral vectors are minimized.^5^ Our study aimed to derive iPSCs from peripheral blood T-cells of DMD patients using episomal plasmid in a feeder free and xeno free cell culture system followed by their characterization using immunofluorescence. The derivation of iPSCs through this approach will open many avenues for future therapeutic applications.

## METHODS

The study was started after getting approval from the Institutions Review Board of Dow University of Health Sciences (IRB-771/DUHS/Approval/2016/293, Dated: September 21^st^, 2016). Informed consent was signed by patient’s guardian before sample collection. The study was carried out at Dow Research Institute of Biotechnology and Biomedical Sciences during August 2017- July 2018 for a period of approximately 12 months.

### Sample Collection

The study participants were recruited from various hospitals and rehabilitation centers in Karachi. Patients between 2-20 years of age with clinical suspicion of DMD, raised CPK levels and myopathic potential on EMG were included in the study.

### Isolation of peripheral blood mononuclear cells from peripheral blood

Peripheral blood mononuclear cells (PBMNC) were separated from whole blood by density gradient centrifugation. PB was mixed with Phosphate buffered saline (PBS, AM9625, Invitrogen) in equal quantity and layered very slowly on ficoll (GE17-5442-02, GE health care) in ratio 1:1:1. The sample was then centrifuged (Eppendorff, 5810R) at 800g for 20 min without brakes at room temperature. This resulted in layers of separation having PBMNCs. This was followed by another round of centrifugation for washing. The cell pellet containing PBMNC was resuspended in complete AIM-V media (12055-091, Invitrogen). The AIM-V media has been formulated for the growth of T cells and contains L- glutamine, streptomycin sulfate at 50 µg/ ml and gentamycin sulfate at 10 µg/ml. Cell viability analysis of PBMNC suspension was done using cell viability analyzer (Beckman Coulter Vi Cell XR).

### Culture of PBMNC to derive T cells

PBMNC were grown in culture at 5 % CO_2_ at 37^o^C in incubator (New Burnswick Galaxy 170 R CO_2_ Incubator) for five days for T cell derivation. Approximately, 10x 10^5^ cells were cultured in freshly prepared AIM-V media (12055-091, Invitrogen) with 300 IU/ml rhIL-2 (10799068001, Sigma Aldrich) and 10ng/ml plate bound anti CD3 antibody (OKT-3 clone, 16-0037-81, eBioscience)^6^. The cells were observed daily under microscope (Leica DMI1 microscope, LAS v 4.6.1) and allowed to grow in the above mentioned culture condition for 5 days. On day 5, T cells were characterized by flow cytometry and then used for reprogramming.

### Characterization of T-cells using flow cytometer

Immunophenotyping of PBMNC derived T cells was done on day 5 using B.D flow cytometer (B.D FACS Celesta, FACS Diva software version-8). Approximately 1X10^6^ PBMNC derived T cells were used for immunophenotyping. CD3 antibody (561800, BD Pharmingen) was used as a positive marker and CD31 antibody (560984, BD Pharmingen) was used as a negative marker for characterization of PBMNC derived T cells.

### DMD patient specific iPSCs generation and culture

Approximately 2.5 x 10^5^ T- cells were transfected with Epi5 episomal reprogramming vectors (A15960, Invitrogen) using neon transfection system (MPK5000, Thermo Fisher Scientific). Post transfection the sample was transferred into a vitronectin coated culture plate containing pre warmed complete AIM-V media. Post transfection microscopy was done and images were captured. The plate was kept in 5 % CO_2_ incubator at 37^o^C. The next day media was switched to N2B27 media containing DMEM/F12 (11330-032, Life technologies), N2 supplement (17502-048, Life technologies), B27 supplement (17504-044, Life technologies), MEM Non- Essential Amino Acid (11140-050, Life technologies), GlutaMAX-I (35050-061, Life technologies), Β-Mercaptoethanol (21985-023, Life technologies) with freshly added 100 ng/ml bFGF (PHG0264, Life technologies). From day 2 post transfection N2B27 media was changed every day until day 8 post transfection and then replaced with Complete E8 media (A1517001, Gibco) every day until the iPSC colonies grew to an appropriate size for transfer. The Complete E8 media consists of Essential 8 Basal medium and E8 supplement (A1517001, Gibco).

### Characterization of iPSC colonies using Immunofluorescence

The iPSC colonies were fixed using a combination of Acetone & Methanol (1:1), blocked in 2 % BSA (15260037, Gibco) in PBS for 30 minutes. Cells were stained with primary antibodies provided in ES cell marker kit (SCR002, Chemicon). Primary antibodies included OCT4 (MS x OCT4, IgG, clone 10H11.2, Chemicon, USA), SSEA4 (MS x SSEA-4, IgG, clone MC-813-70, Chemicon, USA) and TRA-1-81 (MS x TRA-1-81, IgM, clone TRA-1-81, Chemicon, USA). Primary antibody dilutions were made in blocking solution in the ratio of 1: 50 and dispensed onto the marked iPSC colony. The primary antibodies were incubated for one hour at room temperature in the dark. After incubation with primary antibody washing was done thrice with 1X rinse buffer 10 minutes each. This was followed by incubation with secondary antibodies. The secondary antibody used for OCT4 and SSEA4 was FITC labelled anti human IgG secondary antibody (goat) (AF102-0115, Euroimmun US) and the secondary antibody used for TRA-1-81 was Texas Red goat anti mouse IgM secondary antibody (NBP1-73626, Novas). The fixed and stained iPSC colonies were visualized using inverted fluorescent microscope Leica DMI-8 (LAS v 4.8) and images captured.

## RESULTS

### Culture of PBMNC to derive T cells

**Fig.1:**
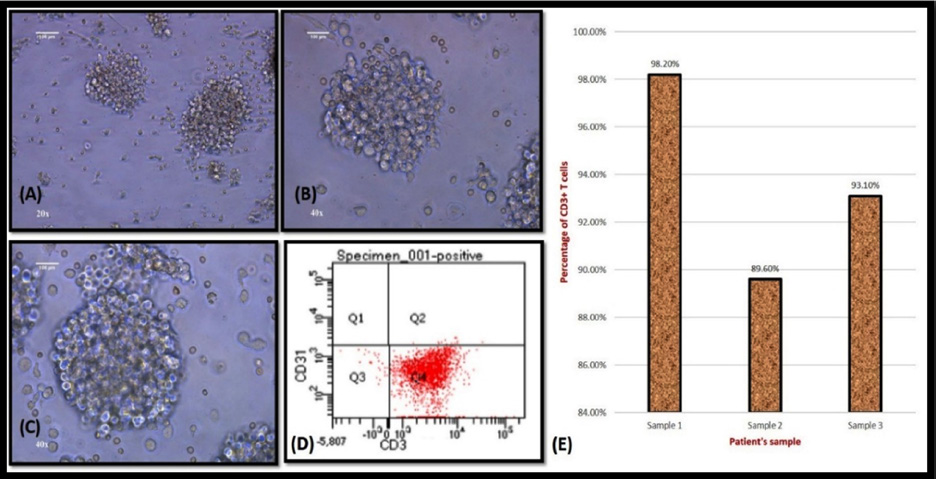
showing the morphology of PBMNC derived T cells using Leica DMi1 Inverted phase contrast microscope, immunophenotyping results using flowcytometry and the percentage of CD3^+^ T cells in n = 3 samples. (A) T cell morphology at day 5 post plating (20 X) (B) T cell morphology at day 5 post plating (40 X). (C) T cell morphology at day 5 post plating (40 X). Scale bar indicates 100 µm. (D) The dot plot shows that the PBMNC derived T cells were positive for the expression of CD3 and negative for the expression of CD31. (E) The percentage of CD3^+^ T cells in n = 3 samples.

Under specific culture conditions as mentioned above the T cells enlarged and formed clumps. By day 3 visible T cell clumps were seen. Morphologically, the T cell clumps appeared as suspended cell clumps and were weakly attached to the bottom of the plate (Fig.1 A-C). Inverted phase contrast microscopy using Leica DMi1 was done every day to assess the growth and proliferation of T cells. The number of T cell clumps increased during the next two days. On day 5, the number of T cell clumps were counted and used for downstream experiments.

### Characterization of PBMNC derived T- cells using flow cytometry

The PBMNC derived T cells were found to be positive for the expression of CD3 and negative for the expression of CD31 (Fig.1 D). By day 5, the percentage of CD3 positive T cells had increased significantly. The percentage of CD3^+^ T cells was 98.2% in sample 1, 89.6% in sample 2 and 93.1% in sample 3. The average percentage of CD3+ T cells was between 89.6% - 98.2% in n = 3 samples (Fig.1 E).

### DMD patient specific iPSCs generation and culture

**Fig.2:**
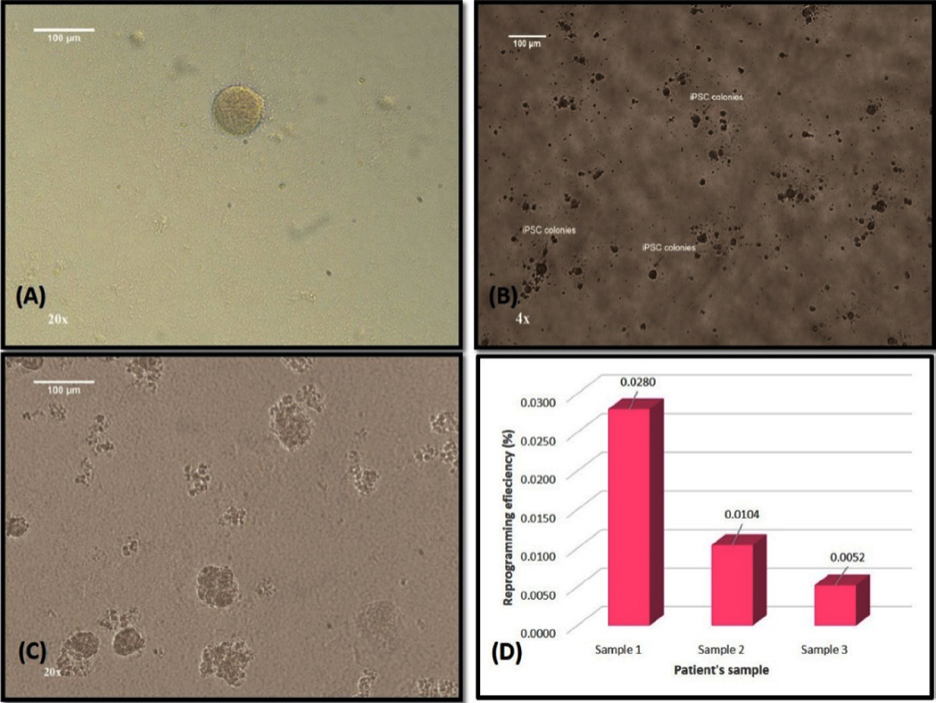
Showing characteristic morphology of human T cell derived iPSCs using Leica DMi1 Inverted phase contrast microscope and reprogramming efficiency in n=3 samples. (A) iPSC colonies at 20X magnification. (B) Representative images of human iPSCs morphology after colony picking 4X and (C) 20X respectively. Scale bar 100 µm. (D) Reprogramming efficiency in n = 3 samples.

The iPSCs resemble embryonic stem cells in morphology. The iPSCs appeared as round, compact and flat colonies with well-defined edges (Fig.2 A). The cellular features include small, round cells with high nucleus: cytoplasm ratio, prominent nucleoli and scant cytoplasm. The iPSCs colonies first appeared on D27 post transfection in sample 1. In sample 2, iPSCs colonies were first seen on D16 post transfection. However, in sample 3, iPSCs colonies were seen on D9 post transfection. Therefore, in n = 3 samples, the iPSCs colony appearance varied from D9- D27 post transfection. The scoring of iPSC colonies was not done as it was not required. However, the reprogramming efficiency was calculated as shown in Figure 2D by dividing all the iPSCs colonies appeared by the number of cells transfected. In n = 3 samples, the average reprogramming efficiency was 0.016 % which is consistent with the literature (Fig.2 D). The iPSCs colonies that were picked were all undifferentiated. E8 media was used specifically for this purpose, so that the iPSCs do not differentiate and can be expanded in undifferentiated state.

### Characterization of DMD patient specific iPSCs through immunofluorescence

**Fig.3:**
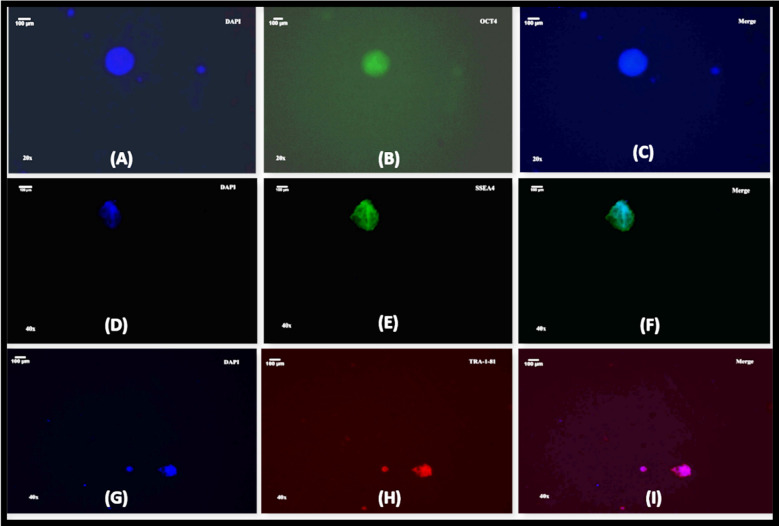
showing immunofluorescence staining depicting protein expression of pluripotency markers in iPSCs colonies (A) DAPI used to stain nuclei (blue). (B)The iPSCs colonies were stained positive for pluripotency markers OCT4, (green). (C) merge image for OCT4 staining.(D) DAPI used to stain nuclei (blue). (E)The iPSC colonies were stained positive for pluripotency markers SSEA4 (green). (F) merge image for SSEA4 staining. (G) DAPI used to stain nuclei (blue). (H)The iPSCs colonies were stained positive for pluripotency marker TRA-1-81 (red). (I) merge image for TRA-1-81 staining. Scale bar indicates 100µm.

The T cell derived iPSCs colonies were positive for the expression of pluripotency markers OCT4, SSEA4 and TRA-1-81. DAPI was used as a nuclear stain to stain the nucleus (Fig.3 A-I).

## DISCUSSION

The patient specific iPSCs are a new therapeutic option for patients as they can escape immune surveillance and are a better therapeutic alternative to embryonic stem cells. The primary goal of our research was to generate DMD patient specific iPSCs with a hope to utilize these iPSCs and its derived cells for future personalized medical remedies.

Peripheral blood T cells have been labeled as a most convenient, suitable, minimally invasive approach to generate iPSCs as compared to other cell sources. It would increase the likelihood of patients giving consent for sampling due to minimal sample requirement. We successfully isolated sufficient T cells from only five ml peripheral blood of DMD patients and reprogrammed these T cells to iPSCs. The ease of in vitro proliferation makes it an ideal cell source for reprogramming In contrast to it, skin fibroblast and dental pulp cells take around 3-4 weeks to establish primary culture.^6^ Considering these factors we have used a better alternative to generate iPSCs as compared to other cell sources. Peripheral blood T lymphocytes of healthy individuals have been used to generate iPSCs using sendai virus.^7^ Induced pluripotent stem cells have been generated from peripheral blood mononuclear cells (PBMNC) using non integrating Sendai virus in normal individuals.^8^

As per our literature review, there have been no studies on derivation of human iPSCs in Pakistan. One study identified deletions in dystrophin gene of Pakistani DMD patients 9 In another study mutation analysis was performed on 2 families in Baluchistan to identify mutations in the distal hotspot region of DMD gene using Sanger sequencing 10_._ Retrospective studies have been conducted on dystrophinopathy patients at Aga Khan University Hospital Karachi utilizing MLPA to screen dystrophin gene exons for deletions/ duplications.^11^,^12^ But no study so far has focused on derivation of iPSCs from these patients for future invitro disease modeling.

EBNA1 based episomal vectors have been used for reprogramming different cell types including fibroblasts^13^_,_ urinary epithelial cells^14^_,_ amniotic fluid cells^14^_,_ CD34^+^ cells^15^_,_ adult peripheral blood mononuclear cells^16^,^17^_,_ dental pulp cells^18^ and T cells.^6^,^19^

To overcome this issue we have generated iPSCs in a feeder free and xeno free culture conditions. We have utilized human recombinant vitronectin and E8 medium for generation of iPSCs eliminating the need for feeder cells and animal derived proteins. Vitronectin is a xeno free cell culture matrix that provides a defined surface for feeder free culture of human pluripotent stem cells.^16^,^20^

The criteria for characterization of iPSCs is based upon their morphology and expression of pluripotency markers. The iPSCs appear as round, compact and flattened tightly packed colonies containing cells with high nucleus to cytoplasmic ratio. The colonies have distinct borders and sharp edges.^3^,^15^,^17^ The derived iPSCs should be positive for the expression of pluripotency markers like OCT4, SOX2, NANOG, LIN28, SSEA-3, SSEA-4, TRA-1-60.^21^ The iPSCs derived by the episomal reprogramming of T cells from Pakistani Duchenne Muscular dystrophy patients were morphologically similar to the previously published data.

### Limitation of the Study

For characterization of PBMNC derived T cells antibodies other than CD3 antibody could have been used but due to time and budget constraints it was not possible.

## CONCLUSION

We were successful in developing a baseline strategy for deriving DMD patient specific iPSCs from peripheral blood T cells of DMD patients using a feeder free and xeno free cell culture system. The method will be helpful in future for developing clinical grade iPS cell lines for therapeutic application in patients suffering from DMD and other diseases. The iPSCs models can also be used for drug screening to develop personalized therapy for each patient in future.

### Author’s Contribution:

**MW;** Designed and supervised the MPhil Student project. All work including cell culture, Flowcytometry, Cellular reprogramming using Episomal Plasmids and immunofluorescence was done under his supervision.

**MZ:** MPhil student who carried out the experimental work under supervision.

**JA:** Co-supervisor for the MPhil Project.
